# Fish Consumption and Coronary Heart Disease: A Meta-Analysis

**DOI:** 10.3390/nu12082278

**Published:** 2020-07-29

**Authors:** Bo Zhang, Ke Xiong, Jing Cai, Aiguo Ma

**Affiliations:** Institute of Nutrition and Health, School of Public Health, Qingdao University, Qingdao 266071, China; zhangzhang19940516@163.com (B.Z.); kexiong@qdu.edu.cn (K.X.); jingfox@163.com (J.C.)

**Keywords:** fish, coronary heart disease, incidence, mortality, systematic review, meta-analysis

## Abstract

Epidemiological studies on the impact of fish consumption on coronary heart disease (CHD) incidence have shown inconsistent results. In addition, in terms of CHD mortality, although previous meta-analyses showed that fish consumption reduces the risk of CHD, six newly incorporated studies show that fish consumption has no impact on CHD. Therefore, the results still need to be verified. The purpose of this study is to quantitatively evaluate the impact of fish consumption on CHD incidence and mortality. Relevant studies were identified from PubMed, Web of Science, and Embase databases up to October 2019. The multivariate-adjusted relative risks (RRs) for the highest versus the lowest fish consumption categories and the 95% confidence intervals were computed with a random-effect model. A restricted cubic spline regression model was used to assess the dose–response relationship between fish consumption and CHD incidence and mortality. Forty prospective cohort studies were incorporated into research. Among them, 22 studies investigated the association between fish consumption and CHD incidence (28,261 cases and 918,783 participants), and the summary estimate showed that higher fish consumption was significantly associated with a lower CHD incidence [RR: 0.91, 95% CI: (0.84, 0.97); *I*^2^ = 47.4%]. Twenty-seven studies investigated the association between fish consumption and CHD mortality (10,568 events and 1,139,553 participants), and the summary estimate showed that higher fish intake was significantly associated with a lower CHD mortality [RR: 0.85, 95% CI: (0.77, 0.94); *I*^2^ = 51.3%]. The dose–response analysis showed that the CHD incidence and mortality were reduced by 4%, respectively, with a 20 g/day increment in fish consumption. This meta-analysis indicates that fish consumption is associated with a lower CHD incidence and mortality.

## 1. Introduction

Coronary heart disease (CHD) remains a major cause of mortality throughout the world [1]. About 17.8 million people died from cardiovascular disease worldwide in 2017. Among them, 8.9 million people were killed by CHD [2]. Many studies have shown that hypertension, diabetes, unhealthy diet, dyslipidemia, abdominal obesity, smoking, and psychosocial stress are the major risk factors for CHD [3,4]. In addition to preventing coronary heart disease by reducing these risk factors, the potential role of dietary factors has also received increasing attention. At the same time, many studies in recent years have shown that adherence to healthier dietary patterns is helpful for CHD prevention [5].

Fish is a kind of food that is rich in omega-3 long-chain polyunsaturated fatty acids (n-3 LCPUFAs) [6]. Many studies have shown that fish consumption may help to reduce the risk of myocardial infarction, hypertension, atherosclerosis, and stroke [7,8,9]. Earlier epidemiological studies found that Alaskan Natives and Greenland Eskimos who eat large amounts of fish have lower levels of mortality from CHD [10,11]. N-3 LCPUFAs have anti-inflammatory and triglyceride lowering effects, and also might have vasodilator, anti-arrhythmia, and anti-hypertension effects [12]. In addition, fish can provide protein, vitamin D, vitamin B, calcium, selenium, and other nutrients [13]. Randomized controlled trials showed that supplementation with L-arginine reduces blood pressure [14] and may allow vascular endothelial function to be recuperated [15]. In addition to their individual roles, synergistic effects among nutrients may have important effects [16,17].

In fact, there have been several meta-analyses of prospective studies about CHD incidence or mortality, and the results remain inconsistent [18,19,20,21]. Whelton et al. [18] found that fish consumption reduced the risk of CHD, but Bechthold et al.’ [21] s research showed that there was no significant association between fish and CHD incidence. Three meta-analyses of cohort studies reported a significant inverse association between fish consumption and CHD mortality [18,19,20]. However, in recent years, many cohort studies have showed no correlation between fish consumption and CHD, and the meta-analysis of cohort studies has not been updated in recent years. Consequently, we conducted an updated meta-analysis to clarify the association between fish consumption and the CHD incidence and mortality.

## 2. Materials and Methods

### 2.1. Search Strategy and Inclusion Criteria

A systematic literature search was performed in the Web of Science, Embase, and PubMed databases until October 2019 using the search query (cardiovascular disease OR coronary heart disease OR myocardial infarction OR CHD) AND (fish OR fish oil OR seafood OR omega-3 fatty acids OR n-3 fatty acid OR polyunsaturated fatty acid). The search was carried out in the titles and abstracts of articles. In addition, a manual search was implemented by screening the reference lists of the original studies, meta-analyses, and reviews, and by using Google and Baidu Scholar.

The inclusion criteria were as follows: (1) prospective cohort studies; (2) fish intake as the exposure; (3) CHD incidence or mortality as the outcome; (4) the use of a general adult population, aged 18 years and older; and (5) inclusion of the estimated relative risks (RRs) and 95% confidence intervals (CIs).

### 2.2. Data Extraction and Quality Assessment

Data extraction was conducted by two investigators (B.Z. and K.X.) independently, and any discrepancies were resolved via discussion to reach a consensus. The basic information about each eligible study was extracted, including the surname of the first author, publication year, country, age, gender, number of cases, sample size, duration of follow-up, fish intake category, fish intake assessment method, outcome assessment method, relative risk (RR), with the corresponding 95% confidence interval (CI), and adjustment covariates. If the lowest intake group was the reference group in the article, we directly extracted the RR value. If the lowest intake group was not the reference group in the article, we did not convert the RR value but instead extracted the converted RR value in accordance with Zhong et al. [22]. In the included articles, the unit of fish intake was servings/day, times/day, or grams/day. We converted servings/day and times/day into grams/day when calculating the dose–response relationship. If the article did not mention the number of per serving fish consumed, we used 105 g for each serving in accordance with He et al. [19].

The Newcastle-Ottawa Scale criteria was used for the quality assessment. The criteria included nine aspects with a maximum of 9 points. Scores of 0–3, 4–6, and 7–9 points indicated low, medium, and high quality, respectively [23].

### 2.3. Statistical Analysis

Multivariate-adjusted RRs with corresponding 95% CIs for the highest versus lowest fish consumption categories were logarithm transformed, and the pooled RR was calculated by using a random-effect model [24]. If the study reported results for the male and female subgroups separately, we combined them using a fixed-effects model and used the combined effect size for the meta-analysis. Heterogeneity among studies was evaluated by *I*^2^ with 25%, 50%, and 75% as cut-off points for low, medium, and high heterogeneity [25]. To identify the source of heterogeneity among the studies, we performed subgroup and meta-regression analyses.

A two-stage, random-effect, dose–response analysis was used to assess a potential non-linear correlation between fish intake and CHD incidence and mortality. A restricted cubic spline model was applied, and the curve trend was obtained by modeling the three knots of the distribution percentile (25%, 50%, and 75%) of fish consumption [26]. By testing the null hypothesis that the regression coefficient of the second spline was equal to zero, the *p* value of the curvilinear association was obtained [27]. In the case of a linear trend (*p* > 0.05), a linear dose–response trend estimation was applied using the generalized least squares regression method proposed by Greenland and Longnecker and Orsini et al. [28,29]. The median or mean for each category of fish consumption was extracted. If the median or mean was not mentioned in the article, we used the midpoint of the upper and the lower boundaries. The intake was defined as 1.2 times the highest boundary if the highest quantile was open-ended. Meanwhile, the fish intake of the lowest quantile (the reference) was set to zero in each study [30]. We performed a sensitivity analysis, deleting one study at a time and recalculating the summary estimate to explore the impact of each study on the summary RR. Publication bias was assessed using Egger’s test (*p* < 0.1 was considered significant) [31]. A statistical analysis was performed using STATA 11.0 (STATA CORP, College station, TX, USA). The *p* value was two-tailed, and the significance level was 0.05.

## 3. Results

### 3.1. Literature Search and Study Characteristics

**Figure 1:**
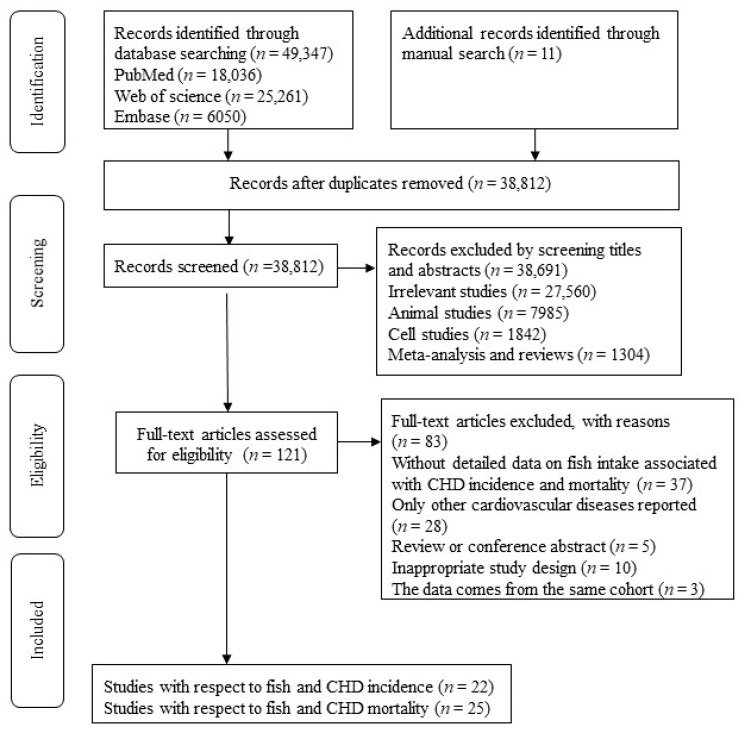
The flow chart for detailed steps of literature search.

The flow chart of the literature search is shown in Figure 1. We identified 18,036 publications from PubMed, 25,261 from the Web of Science, 6050 from Embase, and 11 from the manual search. There were 38,812 articles left after eliminating duplicates. After screening titles and abstracts, we excluded 38,691 articles. By reviewing the full text, we removed 83 articles. Ultimately, we included 38 articles that met the inclusion criteria.

The basic information from the eligible studies is listed in Table 1 and Table 2. The article by Oomen et al. [32] included three cohorts from Italy, Finland, and the Netherlands. In summary, 22 prospective cohort studies were included in the meta-analysis of CHD incidence (28,261 events and 918,783 participants) [33,34,35,36,37,38,39,40,41,42,43,44,45,46,47,48,49,50,51,52,53,54], and 27 prospective cohort studies were included in the meta-analysis of CHD mortality (10,568 events and 1,139,553 participants) [32,34,35,37,38,39,40,42,48,51,55,56,57,58,59,60,61,62,63,64,65,66,67,68,69]. Of these, 18 studies (16 publications) were from Europe [32,39,41,42,44,45,46,48,51,52,53,55,58,62,67,69], 6 studies (6 publications) were from Asia [40,59,61,63,66,68], 16 studies (15 publications) were from America [33,34,35,36,37,38,43,47,49,50,54,56,57,60,65], 1 study (1 publication) was from Australia [64], and 1 study (1 publication) was from Canada [65]. The period of follow-up ranged from 4 to 30 years. According to the Newcastle-Ottawa scale criteria (Tables S1 and S2), 25 studies (23 publications) [32,35,36,37,40,41,42,44,47,48,49,51,52,53,55,56,57,59,60,61,62,66,69] were rated as being of high quality, and 15 studies (15 publications) [33,34,38,39,43,45,46,50,54,58,63,64,65,67,69] were rated as being of medium quality.

### 3.2. Fish Intake and CHD Incidence

**Figure 2:**
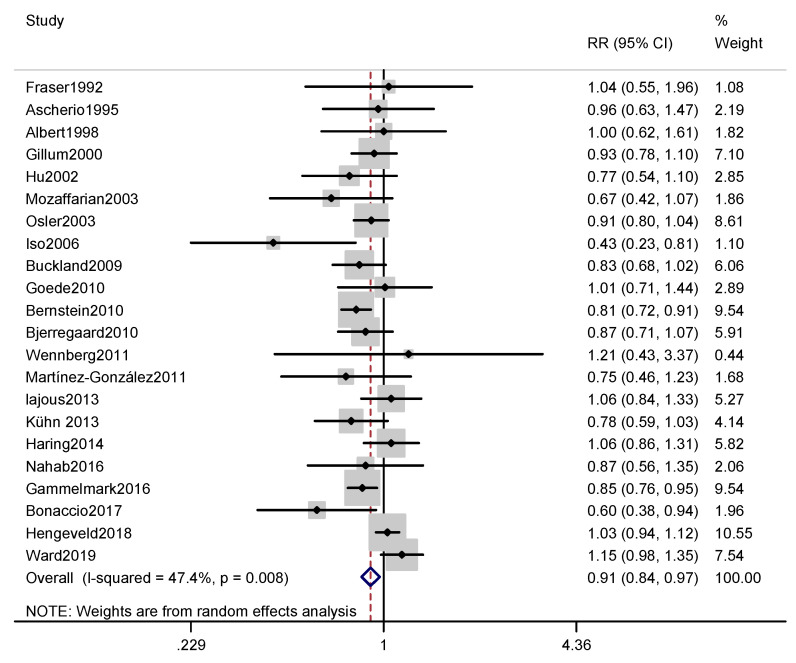
Forest plot of fish consumption with CHD incidence. The pooled effect was calculated by using a random-effects model. The diamond denotes summary risk estimate, and horizontal lines represent 95% CI. Abbreviations: RR, relative risk; CI, Confidence interval.

According to the included articles, the association between fish intake and CHD incidence is shown in Figure 2. By comparing the highest and lowest fish consumption categories, the combined RR for the CHD incidence was found to be 0.91 (0.84, 0.97) (*p* = 0.008), with a moderate level of heterogeneity (*I*^2^ = 47.4%).

### 3.3. Fish Intake and CHD Mortality

**Figure 3:**
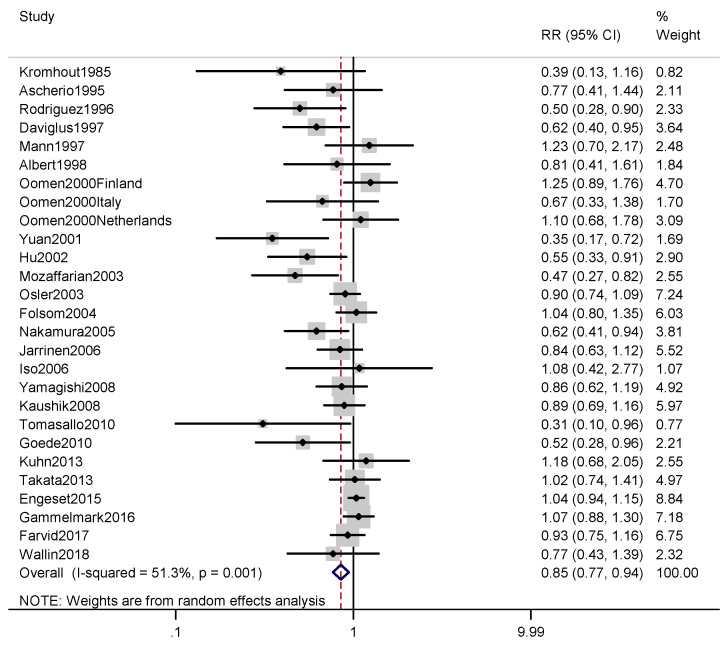
Forest plot of fish consumption with risk of CHD death. The pooled effect was calculated by using a random-effects model. The diamonds denote summary risk estimate, and horizontal lines represent 95% CI. Abbreviations: RR, relative risk; CI, Confidence interval.

According to the included articles, the association between fish intake and the risk of CHD death is shown in Figure 3. By comparing the highest and lowest fish consumption categories, the combined RR for the CHD mortality was found to be 0.85 (0.77, 0.94) (*p* = 0.001), with a moderate level of heterogeneity (*I*^2^ = 51.3%).

### 3.4. Subgroup Analysis and Meta-Regression

The subgroup analysis was performed by region, gender, follow-up period, and article quality (Tables S3 and S4). In terms of CHD incidence, the subgroup analysis showed a significant correlation between fish consumption and CHD incidence in Europe (RR, 0.89; 95% CI, 0.82–0.97; *I*^2^ = 42.2%), but not in North America. In addition, there was a significant correlation between fish consumption and CHD incidence in the female subgroup (RR, 0.85; 95% CI, 0.78–0.92; *I*^2^ = 5.6%), the subgroup with a follow-up period of ≥ 10 years (RR, 0.91; 95% CI, 0.84–0.99; *I*^2^ = 51.5%), and the subgroup with an article quality of ≥ 7 points (RR, 0.90; 95% CI, 0.83–0.98; *I*^2^ = 50.6%). It was not found in the male subgroup, the subgroup with a follow-up period of <10, and the subgroup with an article quality of <7.

In terms of CHD mortality, no significant associations were found in the male subgroup or the female subgroup. In the subgroup analysis by region, a significant association was identified for fish consumption and CHD mortality in the North American studies (RR, 0.65; 95% CI, 0.49–0.86; *I*^2^ = 54.2%), but there was no significant correlation in the studies conducted in Europe and Asia. In addition, a significant association was found in the subgroup with a follow-up period of 10–20 years (RR, 0.88; 95% CI, 0.79–0.99; *I*^2^ = 48.7%) and in the subgroup with high-quality articles (RR, 0.78; 95% CI, 0.66–0.92; *I*^2^ = 55.3%). In addition, as shown in Figure 2, since a level of moderate heterogeneity was found in the analysis of fish consumption and CHD incidence, a meta-regression was performed to explore potential sources of heterogeneity with the covariates of publication year (*p* = 0.324), continent (*p* = 0.132), sex (*p* = 0.102), follow-up period (*p* = 0.734), method of evaluating fish consumption (*p* = 0.711), adjustment (or not) for BMI (*p* = 0.214), and adjustment (or not) for alcohol (*p* = 0.226). However, none of these covariates were found to significantly affect the between-study heterogeneity. Similarly, as shown in Figure 3, since a level of moderate heterogeneity was found in the analysis of fish consumption and CHD mortality, a meta-regression was performed to explore potential sources of heterogeneity with the covariates of publication year (*p* = 0.176), continent (*p* = 0.294), sex (*p* = 0.826), follow-up period (*p* = 0.995), method of evaluating fish consumption (*p* = 0.094), adjustment (or not) for BMI (*p* = 0.970), adjustment (or not) for alcohol (*p* = 0.900). However, none of these covariates significantly affected the between-study heterogeneity.

### 3.5. Dose–Response Relationship

**Figure 4:**
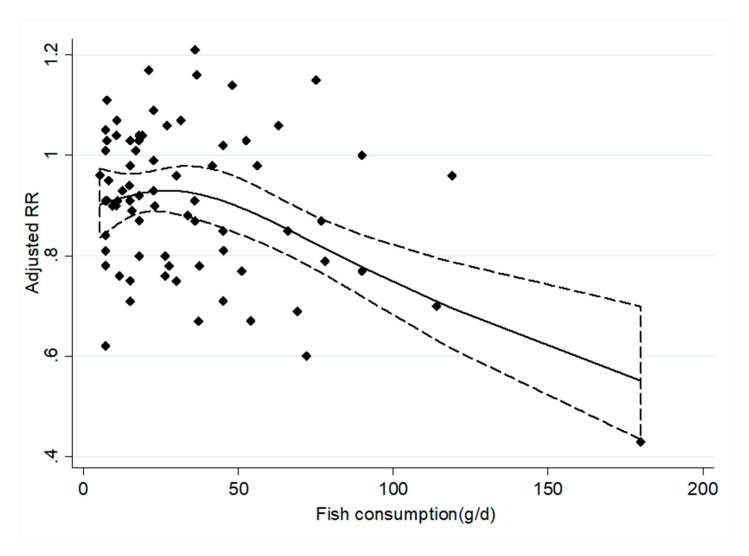
The dose–response analysis between the fish consumption and the CHD incidence. The diamonds represented the adjusted RRs for each exposure quantile of fish consumption in the included individual studies. The solid line and the long dash line represent the estimated relative risk (RR) and its 95% confidence interval (CI). Abbreviations: g/d, grams per day.

**Figure 5:**
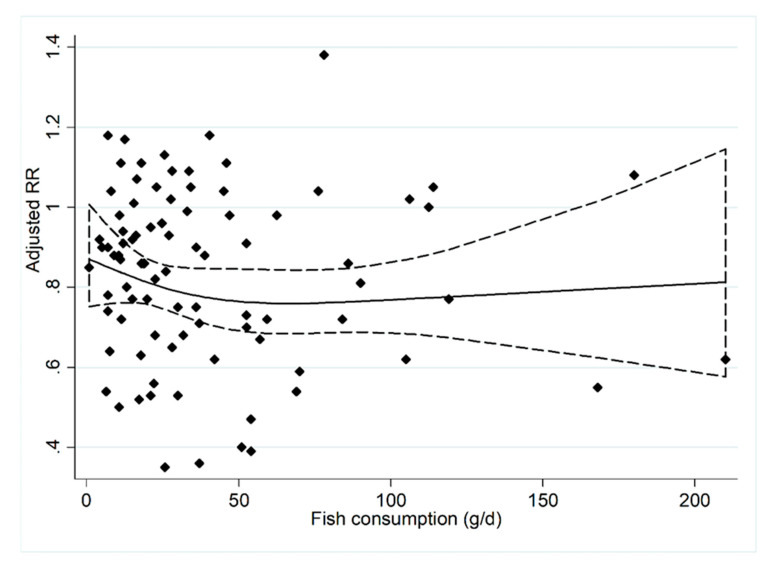
The dose–response analysis between the fish consumption and the CHD mortality. The diamonds represented the adjusted RRs for each exposure quantile of fish consumption in the included individual studies. The solid line and the long dashed line represent the estimated relative risk (RR) and its 95% confidence interval (CI). Abbreviations: g/d, grams per day.

Nineteen prospective cohort studies [33,34,35,36,37,38,39,40,42,43,44,45,48,49,50,51,52,53,54] were included in the dose–response analysis concerning fish consumption and CHD incidence (Figure 4). The results showed that an increase in fish intake by 20 g/d is associated with a 4% [RRs = 0.96, 95% CI: (0.95, 0.97)] reduction in CHD incidence. Twenty-three prospective cohort studies [32,34,35,37,38,39,40,42,48,51,55,57,59,60,61,62,63,66,67,68,69] were included in the dose–response analysis concerning fish consumption and CHD mortality (Figure 5). The results showed that an increase in fish intake by 20 g/d is associated with a 4% [RRs = 0.96, 95% CI: (0.95, 0.98)] reduction in CHD mortality.

### 3.6. Sensitivity Analysis and Publication Bias

The results of the sensitivity analysis are shown in Figures S1 and S2. Each article was excluded in order, and the summary results did not change significantly. In the publication bias analysis, when analyzing the relationship between fish intake and CHD incidence, the Egger’s test indicated that no evidence of publication bias was found (*p* = 0.226). Besides, publication bias was also not observed through visual inspection of the funnel plot (Figure S3). When analyzing the relationship between fish intake and CHD mortality, the Egger’s test revealed that there may have been a publication bias (*p* < 0.001). In addition, by observing the funnel plot (Figure S4), there was found to be a publication bias. However, after using the trim and fill method, no trimming was performed.

## 4. Discussion

The results of this meta-analysis showed that higher fish consumption is inversely related to CHD incidence and mortality. In terms of CHD incidence, our result is consistent with Whelton et al.’s results [18]. Compared with the review by Bechthold et al. [21], our review excluded four studies. The reasons were as follows: two studies did not provide RR values for fish consumption and CHD incidence [70,71], one study reported fish and shellfish consumption rather than pure fish consumption [72], and the last one did not provide fish consumption data [73]. In terms of CHD mortality, although the six newly incorporated studies all showed that there is no association between fish consumption and CHD mortality, the results of our review are consistent with previous reviews [18,19,20] and also confirm the findings presented in previous reviews.

The dose–response analysis showed that the CHD incidence and mortality were both reduced by 4%, with a 20 g/day increment in fish consumption. As shown in Figure 4 and Figure 5, when fish consumption was above 40 g/day, the risk of CHD development decreased as intake increased. When fish consumption was below 60 g/day, the risk of CHD death decreased as intake increased. Therefore, we believe that 60 g/d is the ideal dose for preventing CHD mortality. This is basically consistent with the average fish intake of the population of Japan [74]. However, the average intake of people in China and Europe is lower than this level [75,76]. The findings of our study have important clinical and public health implications for primary and secondary prevention of CHD.

In this meta-analysis, between-study heterogeneity was investigated in the analysis of fish intake and CHD incidence and mortality. To discover the potential sources of heterogeneity, we performed a meta regression. The results showed that no covariates (publication year, continent, sex, evaluation method of fish consumption, follow-up period, adjustment for BMI, and adjustment for alcohol) had a significant effect on the heterogeneity between studies. Then, a subgroup analysis was conducted. In the subgroup analysis of fish and CHD incidence, the heterogeneity of the male and female subgroups was reduced to 3.9% and 5.6%, respectively. Gender was found to contribute to the heterogeneity. The reason for this phenomenon may be that women’s depletion of estrogen during menopause increases the risk of developing CHD [77]. Higher levels of total testosterone in men are associated with lower risk of developing CHD [78,79]. Testosterone has anabolic effects, namely, the promotion of muscle mass and strength [80]. Carlos et al. found that higher grip strength was associated with lower risk of morbidity and mortality from cardiovascular disease [81]. We also conducted a sensitivity analysis to reduce the heterogeneity. Each article was excluded in order, and the summary results did not change significantly. Presumably, differences in fish type, fish cooking style, characteristics of the sample, and variability in the adjustment of confounding factors may also influence the heterogeneity.

The potential mechanism for preventing CHD with fish consumption is as follows. N-3 LCPUFA may play an important role in this process, mainly due to its anti-arrhythmic [82,83] and anti-inflammatory properties and because it improves endothelial dysfunction [84,85]. After being incorporated into cell membrane phospholipids, seafood-derived n-3 LCPUFA can produce electrophysiological effects, such as beneficial effects on cardiac ion channel function and cell signaling pathways and increased cell membrane fluidity [86,87]. These effects have been linked to a reduced risk of ventricular arrhythmias [83] and sudden cardiac death [88].

On the other hand, fish is considered an excellent source of protein [89]. Essential amino acids, fat-soluble vitamins [90], and other types of fatty acids [91] in fish may also have effects. For example, fish is a good source of vitamin D. After vitamin D enters the body, it can regulate blood pressure, inhibit inflammation, and inhibit vascular smooth muscle proliferation and vascular calcification [92]. Fish also contains trace elements, including selenium and calcium. Selenium can alleviate oxidative stress and inflammation in patients with CHD [93]. Therefore, eating fish may be more beneficial than supplementing with n-3 fatty acids alone.

Our study has some advantages. At first, our study included more articles and more participants. Because our study primarily considered the highest versus the lowest fish consumption categories, one article excluded in the previous study [20] that included two groups of fish consumption was reincorporated (< 2 times per week versus ≥ 2 times per week) [56]. Two studies that did not use the lowest fish consumption group as the reference group were reincorporated [39,61]. Secondly, the selected studies were cohort studies, including 23 high-quality articles and 15 medium-quality articles. Compared with retrospective studies, the possibility of selection bias and recall errors was reduced. Moreover, most studies had a long follow-up period (12-30 years). CHD is a chronic disease, and a longer follow-up period can better explain the association between fish consumption and CHD. Thirdly, the sensitivity analysis showed that after deleting any one study, there was no significant change in the combined estimates, which indicated the stability of the combined estimates.

At the same time, there are some limitations in this study. First, there were regional differences between studies. Eastern and Western food cultures are different, and there are some differences in fish species and cooking methods, which may have impacted on the results. Secondly, most studies assessed fish consumption through the food frequency questionnaire, in which some inaccurate assessments or records are inevitable. Thirdly, although the results were all adjusted for confounding factors, the confounding factors were different. Fourth, we could not obtain detailed fish consumption measurement methods, and therefore it was difficult to standardize fish consumption. Instead, we compared the highest versus the lowest fish intake groups. Fifth, in the subgroup analysis of this study, especially the regional subgroup analysis, although the data used were accurate and true, conflicting results were produced that we cannot explain clearly. Further ongoing studies with a sufficient sample size, standardized dosing, and adequate follow-up duration are required to clarify the contradictory phenomena.

## 5. Conclusions

In conclusion, our meta-analysis indicates that a higher dietary intake of fish is negatively correlated with CHD incidence and mortality. This finding has important public health implications in terms of the prevention of CHD. Since most of the research was conducted in male groups and Western countries, further research needs to be performed in female groups and other regions.

## Figures and Tables

**Table 1:** Characteristics of cohort studies included in the meta-analysis of fish consumption and coronary heart disease (CHD) incidence.

Study Source (Year), Country	Age (Gender)	Subjects (Cases)	Follow-Up Period	Fish Intake Category	Exposure Measure	Outcome Measure	Covariates Adjusted
Fraser [33] (1992), USA	≥25 (Both)	26,743 (134)	6 y	Never; <1/wk; ≥1/wk	FFQ	Obtained from medical records	Age, sex, smoking, exercise, relative weight, and high blood pressure
Ascherio [34] (1995), USA	40–75 (M)	44,895 (547)	6 y	<1/mo; 1–3/mo; 1/wk; 2–3/wk; 4–5/wk; ≥6/wk	FFQ	Based on medical records and autopsy report	Age, body mass index, smoking habits, alcohol consumption, history of hypertension, history of diabetes, history of hypercholesterolemia, family history of myocardial infarction before 60 y of age and profession
Albert [35] (1998), USA	40–84 (M)	20,551 (308)	11 y	1/mo; 1–3/mo; 1–2/wk; 2–5/wk; ≥5/wk	FFQ	Obtained from hospital medical records	Age, aspirin, -carotene treatment assignment, evidence of cardiovascular disease before 12-mo questionnaire, body mass index, smoking status, history of diabetes, history of hypertension, history of hypercholesterolemia, alcohol consumption, vigorous exercise, and vitamin E, vitamin C, and multivitamin use
Gillum [36] (2000), USA	25–74 (Both)	8825 (2007)	18.8 y	Never; <1/wk; 1/wk; >1/wk	FFQ	ICD9	Age, smoking, history of diabetes, education, high school graduate, systolic blood pressure, serum cholesterol concentration, body mass index, alcohol intake, and physical activity.
Hu [37] (2002), USA	34–50 (F)	84,688 (1029)	16 y	<1/mo; 1–3/mo; 1/wk; 2–4/wk; ≥5/wk	FFQ	Based on medical records, death certificate and autopsy report	Age, time periods, smoking status, body mass index, alcohol intake, menopausal status and postmenopausal hormone use, vigorous to moderate activity, No. of times aspirin was used per week, multivitamin use, vitamin E supplement use, and history of hypertension, hypercholesterolemia, diabetes, intake of trans-fat, the ratio of polyunsaturated fat to saturated fat, and dietary fiber
Mozaffarian [38] (2003), USA	≥66 (Both)	3910 (363)	9.3 y	≤1/mo; 1–3/mo; 1/wk; 2/wk; ≥3/wk	FFQ	Based on medical records and death certificate	Age, gender, education, diabetes, smoking, body mass index, systolic blood pressure, LDL cholesterol, HDL cholesterol, triglycerides, C-reactive protein, saturate fat, alcohol, beef/pork, fruit and vegetables
Osler [39] (2003), Denmark	30–70 (Both)	8497 (491)	11 y	≤1/mo; 2/mo; 1/wk; ≥2/wk	Self-administered questionnaire	ICD-8 (codes 410–414), ICD-10 (codes I20–I25)	Familial predisposition, smoking status, physical activity, alcohol, educational status, healthy diet score, total cholesterol, and body mass index
Iso [40] (2006), Japan	40–59 (Both)	41,578 (196)	11 y	Median intake: 23 g/d; 51 g/d; 78 g/d; 114 g/d; 180 g/d	FFQ	Self-reported (letter, telephone), medical records	Age, sex, cigarette smoking, alcohol intake, body mass index, histories of hypertension and diabetes, medication use for hypercholesterolemia, education level, sports at leisure time, quintiles of dietary intake of fruits, vegetables, saturated fat, monounsaturated fat, n6 polyunsaturated fat, cholesterol, and total energy
Buckland [41] (2009), Spain	29–69 (Both)	40,757 (606)	10.4 y	g/1000 kcal/d: 0–16.9; >16.9–31.0; >31.0–266.7	Self-reported questionnaires	Obtained from hospital medical records	Center and age and were adjusted for education, physical activity, body mass index, smoking status, diabetes, hypertension, and hyperlipidemia status, and total calorie intake.
de Goede [42] (2010), The Netherlands	20–65 (Both)	21,342 (252)	11.3 y	<3.3 g/d; 3.3–7.3 g/d; 7.4–14.0 g/d; >14 g/d	FFQ	ICD-9 (codes 410)	Age, gender, BMI, total energy intake, ethanol intake, cigarette smoking, social economic status, vitamin or mineral supplement use, use of drugs for hypertension or hypercholesterolaemia,
Bernstein [43] (2010), USA	30–55 (F)	84,136 (3162)	26 y	Median servings per day: 0.07; 0.11; 0.14; 0.25; 0.43	FFQ	obtained from the medical records	Age, time period, total energy, cereal fiber, alcohol, trans fat, body mass index, cigarette smoking, menopausal status, parental history of early myocardial infarction, multivitamin use, vitamin E supplement use, aspirin use at least once per week, physical exercise
Bjerregaard [44] (2010), Denmark	50–64 (Both)	54,226 (1122)	7.6 y	Male: 0–24 g/d; 25–35 g/d; 36–47 g/d; 48–64 g/d; >64 g/d; Female: 0–22 g/d; 23–31 g/d; 32–41 g/d; 42–54 g/d; >55 g/d	FFQ	(ICD-8:410.00–410.99 and 427.27; ICD-10: I20.0, I21.0–I21.9, and I46.0–I46.9)	Education, smoking, alcohol intake, body mass index, history of diabetes mellitus, systolic blood pressure, serum cholesterol, physical activity, dietary intake of fruits and vegetables, total energy intake, dietary intake of saturated fat, monounsaturated fat, and polyunsaturated fat
Wennberg [45] (2011), Sweden	30–77 (Both)	930 (431)	12 y	Meals/wk: <1/mo; 1/mo; <1/wk; 1–2/wk; >2/wk	FFQ	Based on medical records	Apolipoprotein B/apolipoprotein A-I, smoking, SBP, diabetes, educational level, consumption of fruit and vegetables
Martínez-González [46] (2011), Spain	Mean age: 38 (Both)	13,609 (68)	4.9 y	Male: <87 g/d; ≥87 g/dFemale: <86 g/d; ≥86 g/d	FFQ	Based on medical records	Age, sex, family history of CHD, total energy intake, physical activity, smoking, BMI, diabetes at baseline, use of aspirin, history of hypertension and history of hypercholesterolemia
Lajous [47] (2013), USA	40–75(M) 30–55(F)	79,569 (3756)	18 y(M) 22 y(F)	0 servings/wk; ≥1 servings/wk	FFQ	Based on medical records	Age, parental history of myocardial infarction, oral contraceptive use, body mass index, smoking, menopausal status, hormone replacement therapy, physical activity, aspirin use, vitamin E supplement use, multivitamin supplement use, high blood pressure, high cholesterol, diabetes, angina or coronary artery bypass grafting, stroke, and intakes of calories, trans-fats, alcohol, cereal fiber, red meat, and fish
Kuhn [48] (2013), Germany	35–65 (Both)	48,315 (488)	8.1 y	<7.5 g/d; 7.5–14.5 g/d; 14.5–21.5 g/d; 21.5–31.1 g/d; >31.1 g/d	FFQ	Medical verification of self-reports of incident disease from questionnaires	Age, gender, energy intake, alcohol intake, BMI, waist circumference, physical activity, educational attainment, smoking and prevalent diabetes mellitus.
Haring [49] (2014), USA	45–64 (Both)	12,066 (1147)	22 y	Servings/d: 0; 0.1; 0.2; 0.3; 0.6	FFQ	Information from study visits, yearly telephone follow-up calls, review of hospital discharge lists and medical charts, death certificates,	Age, sex, race, study center, total energy intake, smoking, education, systolic blood pressure, use of antihypertensive medication, HDLc, total cholesterol, use of lipid lowering medication, body mass index, waist-to-hip ratio, alcohol intake, sports-related physical activity, leisure-related physical activity, carbohydrate intake, fiber intake, and magnesium intake.
Nahab [50] (2016), USA	≥40 (Both)	16,479 (440)	5.1 y	Servings: <1/mo; 1-3/mo; 1–2/wk; ≥2/wk	FFQ	Based on medical records	Age, race, region, sex, income, education, exercise, smoking status, Mediterranean diet score, regular aspirin use and total energy intake, current use of hypertensive medication, diabetes status, SBP, BMI, dyslipidaemia
Gammelmark [51] (2016), men, Danish	50–64 (M)	25,913 (2136)	17 y	0–8 g/d; >8–13 g/d; >13–18 g/d; >18–28 g/d; >28 g/d	FFQ	ICD8 and ICD10	Smoking, BMI, waist circumference, physical activity, alcohol intake, educational level, history of diabetes mellitus, hypercholesterolaemia, hypertension, total energy intake, intake of fruits and vegetables and intake of nuts
Gammelmark [51] (2016), women, Danish	50–64 (F)	28,991 (892)	17 y	0–6 g/d; >6–10 g/d; >10–15 g/d; >15–23 g/d; >23 g/d	FFQ	ICD8 and ICD10	Smoking, BMI, waist circumference, physical activity, alcohol intake, educational level, history of diabetes mellitus, hypercholesterolaemia, hypertension, total energy intake, intake of fruits and vegetables and intake of nuts
Bonaccio [52] (2017), Italy	≥35 (Both)	20,969 (287)	4.3 y	times/wk: 0–1.99; 2–3.99; ≥4	FFQ	ICD9	Age, sex, energy intake, education, smoking, drugs for diabetes, drugs for hypertension, drugs for lipids, MDS without fish, blood glucose, LDL-cholesterol and low-grade inflammation
Hengeveld [53] (2018), The Netherlands	20–70 (Both)	34,033 (2134)	18 y	portion/wk: 0; <1; ≥1	FFQ	ICD10(I20–I25, I46, and R96)	Age, sex, physical activity, smoking status, education level, BMI, alcohol intake, total energy intake, intakes of saturated fatty acids, trans fatty acids, fruit, vegetables, and dietary fibre
Ward [54] (2019), USA	Mean age:66 (Both)	197,761 (6265)	6 y	<1/mo; 1–3/mo; 1/wk; 2–4/wk; ≥5/wk	FFQ	ICD-9 (codes 410)	Age, sex, race, BMI, education, smoking status, alcohol intake, exercise

FFQ, Food-frequency questionnaire; BMI, body mass index; ICD, International Classification of Diseases; F, female; M, male; y, year; HDL, High Density Lipoprotein; LDL, Low Density Lipoprotein; SBP, Systolic Blood Pressure; wk, week; mo, month; d, day.

**Table 2:** Characteristics of cohort studies included in the meta-analysis of fish consumption and CHD mortality.

Study Source (Year), Country	Age (Gender)	Subjects (Cases)	Follow-Up Period	Fish Intake Category	Exposure Measure	Outcome Measure	Covariates Adjusted
Kromhout [55] (1985), The Netherlands	40–59 (M)	852 (78)	20 y	0 g/d; 1–14 g/d; 15–29 g/d; 30–44 g/d; ≥45 g/d	Interview	ICD-8 (codes 410–413)	Age, systolic blood pressure, serum total cholesterol, cigarette smoking, subscapular skinfold thickness, physical activity, energy intake, dietary cholesterol, prescribed diet and occupation
Ascherio [34] (1995), USA	40–75 (M)	44,895 (264)	6 y	<1/mo; 1–3/mo; 1/wk; 2–3/wk; 4–5/wk; ≥6/wk	FFQ	Based on medical records and autopsy report, ICD codes are not available	Age, body mass index, smoking habits, alcohol consumption, history of hypertension, history of diabetes, history of hypercholesterolemia, family history of myocardial infarction before 60 y of age and profession
Rodriguez [56] (1996), USA	45–68 (M)	534 (-)	23 y	<2/wk; >2/wk	Interview	Based on death certificates, supplemented with the State Department of Health	Age, years lived in Japan, total calories/d, alcohol intake, physical activity, years smoked, hypertension, and serum cholesterol, glucose, and uric acid levels for past and current smokers separately
Daviglus [57] (1997), USA	40–55 (M)	1822 (430)	30 y	0 g/d; 1–17 g/d; 18–34 g/d; ≥35 g/d	Interview	ICD-8 (codes 410–414)	Age, education, religion, systolic pressure, serum cholesterol, no. of cigarettes smoked per day, BMI, diabetes, ECG abnormalities, daily intakes of energy, cholesterol, SFA, MUFA, PUFA, total protein, carbohydrate, alcohol, Fe, thiamin, riboflavin, niacin, vitamin C, b-carotene and retinol
Mann [58] (1997), UK	16–79 (both)	10,802 (64)	13.3 y	Never, <1/wk, ≥1/wk	FFQ	ICD-9 (codes 410–414)	Age, sex, smoking and social class
Albert [35] (1998), USA	40–84 (M)	20,551 (308)	11 y	1/mo; 1–3/mo; 1–2/wk; 2–5/wk; ≥5/wk	FFQ	ICD-9 (codes 410–414)	Age, aspirin, -carotene treatment assignment, evidence of cardiovascular disease before 12-mo questionnaire, body mass index, smoking status, history of diabetes, history of hypertension, history of hypercholesterolemia, alcohol consumption, vigorous exercise, and vitamin E, vitamin C, and multivitamin use
Oomen [32] (2000), Finland	50–69 (M)	1088 (242)	20 y	0–19 g/d; 20–39 g/d; ≥40 g/d	Interview based on Burke’s diet history method	ICD-8 (codes 410–414, 795)	Age, body mass index, cigarette smoking, intake of energy, vegetables, fruits, alcohol, meat, butter, and margarine
Oomen [32] (2000), Italy	50–69 (M)	1097 (116)	20 y	0 g/d; 1–19 g/d; 20–39 g/d; ≥40 g/d	Interview based on Burke’s diet history method	ICD-8 (codes 410–414, 795)	Age, body mass index, cigarette smoking, intake of energy, vegetables, fruits, alcohol, meat, butter, and margarine
Oomen [32] (2000), The Netherlands	50–69 (M)	553 (109)	20 y	0 g/d; 1–19 g/d; ≥20 g/d	Interview based on Burke’s diet history method	ICD-8 (codes 410–414, 795)	Age, body mass index, cigarette smoking, intake of energy, vegetables, fruits, alcohol, meat, butter, and margarine
Yuan [59] (2001), China	45–64 (M)	18,244 (187)	12 y	<30 g/wk; 30–<60 g.wk; 60–<100 g/wk; 100–<150 g/wk; ≥150 g/wk	FFQ	ICD-9 (codes 410–414)	Age, total energy intake, level of education, body mass index, current smoker, average no. of cigarettes smoked per day, no. of alcoholic drinks consumed per week, history of diabetes, and history of hypertension
Hu [37] (2002), USA	34–50 (F)	84,688 (484)	16 y	<1/mo; 1–3/mo; 1/wk; 2–4/wk; ≥5/wk	FFQ	Based on medical records, death certificate and autopsy report; ICD codes are not available	Age, time periods, smoking status, body mass index, alcohol intake, menopausal status and postmenopausal hormone use, vigorous to moderate activity, No. of times aspirin was used per week, multivitamin use, vitamin E supplement use, and history of hypertension, hypercholesterolemia, diabetes, intake of trans-fat, the ratio of polyunsaturated fat to saturated fat, and dietary fiber
Osler [39] (2003), Denmark	30–70 (both)	7389 (247)	11 y	≤1/mo; 2/mo; 1/wk; ≥2/wk	Self-administered questionnaire	ICD-8 (codes 410–414)	Familial predisposition, smoking status, physical activity, alcohol, educational status, healthy diet score, total cholesterol, and body mass index
Mozaffarian [38] (2003), USA	≥66 (both)	3910 (247)	9.3 y	≤1/mo; 1–3/mo; 1/wk; 2/wk; ≥3/wk	FFQ	Based on medical records and death certificate. ICD codes are not available	Age, gender, education, diabetes, smoking, body mass index, systolic blood pressure, LDL cholesterol, HDL cholesterol, triglycerides, C-reactive protein, saturate fat, alcohol, beef/pork, fruit and vegetables
Folsom [60] (2004), USA	55–69 (F)	41,836 (922)	14 y	<0.5/wk; 0.5–<1/wk; 1–1.5/wk; <1.5–>2.5/wk; ≥2.5/wk	FFQ	ICD-9 (codes 410–414,429.2) or ICD-10 (codes I20–I25, I51.6)	Age, energy intake, educational level, physical activity level, alcohol consumption, smoking status, pack-years of cigarette smoking, age at first live birth, oestrogen use, vitamin use, BMI, waist:hip ratio, diabetes, hypertension, intakes of whole grains, fruit and vegetables, red meat, cholesterol and saturated fat
Nakamura [61] (2005), Japan	≥30 (both)	8879 (142)	19 y	<1/wk; 1–2/wk; 0.5/d; 1/d; ≥2/d	Self-administered questionnaire	ICD-9 or ICD10	Age, sex, cigarette smoking and alcohol intake, hypertension, body mass index, diabete. serum total cholesterol concentration
Jarvinen [62] (2006), men, Finland	30–79 (M)	2775 (335)	21.5 y	≤11 g/d; 12–21 g/d; 22–35 g/d; 36–62 g/d; ≥63 g/d	Interview	ICD-9 (codes 410–414)	Age, energy intake, area, BMI, serum cholesterol, blood pressure, smoking, occupation and diabetes
Jarvinen [62] (2006), women, Finland	30–79 (F)	2445 (163)	21.5 y	≤8 g/d; 9–15 g/d; 16–24 g/d; 25–40 g/d; ≥41 g/d	Interview	ICD-9 (codes 410–414)	Age, energy intake, area, BMI, serum cholesterol, blood pressure, smoking, occupation and diabetes
Iso [40] (2006), Japan	40–59 (both)	41,578 (62)	11 y	Median intake: 23 g/d; 51 g/d; 78 g/d; 114 g/d; 180 g/d	FFQ	ICD-10(I21–I23, I46, and I50)	Age; sex; cigarette smoking; alcohol intake; body mass index; histories of hypertension and diabetes; medication use for hypercholesterolemia; education level; sports at leisure time; quintiles of dietary intake of fruits, vegetables, saturated fat, monounsaturated fat, n6 polyunsaturated fat, cholesterol, and total energy
Yamagish [63] (2008), Japan	40–79 (both)	57,972 (419)	12.7 y	0–27 g/d; 27–39 g/d; 39–53 g/d; 53–72 g/d; 72–229 g/d	FFQ	ICD 10 (codes I20–I25)	Age, gender, energy, history of hypertension and diabetes mellitus, smoking status, alcohol consumption, BMI, mental stress, walking, sports, education level, total energy, and dietary intakes of cholesterol, saturated and n-6 polyunsaturated fatty acids, vegetables and fruit
Kaushik [64] (2008), Australia	≥49 (both)	2683 (287)	12 y	<1/wk; 1/wk; ≥2/wk	FFQ	Based on the Australian National Death Index (NDI) database	Age, gender, mean arterial blood pressure, body-mass index, smoking status, glucose, cholesterol, white cell count, platelet count, qualification level, self-rated health, past history of myocardial infarction and stroke, total vegetable and fat intake
Tomasallo [65] (2010), USA	Mean 48.5 (both)	940 (26)	12 y	≤1/mo; >1/mo–<1/wk; ≥1/wk	Interview	ICD-9 (codes 410–414) or ICD-10 (codesI20–25)	Sex, age, BMI and income at study baseline
Goede [42] (2010), The Netherlands	20–65 (both)	21,342 (82)	11.3 y	<3.3 g/d; 3.3–7.3 g/d; 7.4–14.0 g/d; >14 g/d	FFQ	ICD-9 (codes 410–414) or ICD-10 (codes I20–I25)	Age, gender, BMI, total energy intake, ethanol intake, cigarette smoking, social economic status, vitamin or mineral supplement use, use of drugs for hypertension or hypercholesterolaemia, family history of CVD, SFA, fruit and vegetables
Kuhn [48] (2013), Germany	35–65 (both)	48,315 (117)	8.1 y	<7.5 g/d; 7.5–14.5 g/d; 14.5–21.5 g/d; 21.5–31.1 g/d; >31.1 g/d	FFQ	ICD-10 (I21)	Age, gender, energy intake, alcohol intake, BMI, waist circumference, physical activity, educational attainment, smoking and prevalent diabetes mellitus.
Takata [66] (2013), China	40–74 (both)	134,296 (476)	11.2 y	median intake: 10.6 g/d; 24.7 g/d; 38.8 g/d; 59.3 g/d; 106.2 g/d	FFQ	ICD-9 (codes 410–414)	Age, total energy intake, income, occupation, education, comorbidity index, physical activity level, red meat intake, poultry intake, total vegetable intake, total fruit intake, smoking history, and alcohol consumption
Engeset [67] (2015), men, Italy; Spain; UK; The Netherlands; Greece; Sweden; Denmark; Norway; Germany; France	35–70 (M)	143,183 (2215)	16 y	median intake:1.9 g/d; 10.8 g/d; 21.1 g/d; 34.2 g/d; 76.2 g/d	FFQ	ICD-10 (I20-25)	Energy from fat, energy from carbohydrates and proteins, dietary fibres, red meat, processed meat, vegetables, fruit, alcohol intake, body mass index, physical activity, smoking, education.
Engeset [67] (2015), women, Italy; Spain; UK; The Netherlands; Greece; Sweden; Denmark; Norway; Germany; France	35–70 (F)	337,352 (1050)	16 y	median intake:1.9 g/d; 10.8 g/d; 21.1 g/d; 34.2 g/d; 76.2 g/d	FFQ	ICD-10 (I20-25)	Energy from fat, energy from carbohydrates and proteins, dietary fibres, red meat, processed meat, vegetables, fruit, alcohol intake, body mass index, physical activity, smoking, education.
Gammelmark [51] (2016), men, Danish	50–64 (M)	25,913 (424)	17 y	0–8 g/d; >8–13 g/d; > 13–18 g/d; >18–28 g/d; >28 g/d	FFQ	ICD-8 (410.00-410.99) or ICD-10 (I21.0-I21.9)	Smoking, BMI, waist circumference, physical activity, alcohol intake, educational level, history of diabetes mellitus, hypercholesterolaemia, hypertension, total energy intake, intake of fruits and vegetables and intake of nuts
Gammelmark [51] (2016), women, Danish	50–64 (F)	28,991 (156)	17 y	0–6 g/d; >6–10 g/d; >10–15 g/d; >15–23 g/d; >23 g/d	FFQ	ICD-8 (410.00-410.99) or ICD-10 (I21.0-I21.9)	Smoking, BMI, waist circumference, physical activity, alcohol intake, educational level, menopausal status, history of diabetes mellitus, hypercholesterolaemia, hypertension, total energy intake, intake of fruits and vegetables and intake of nuts
Farvid [68] (2017), Iran	36–85 (both)	42,403 (762)	11 y	median intake: 0 g/d; 0.85 g/d; 5.1 g/d; 16.15 g/d	FFQ	ICD-10 (I20-52)	Gender; age; ethnicity; education; marital status; residency; smoking; opium use; alcohol; BMI; systolic blood pressure; occupational physical activity; family history of cancer; wealth score; medication; and energy intake.
Wallin [69] (2018), Swedish	45–84 (both)	2225 (154)	14 y	≤3/mo; 1–<2/wk; 2–3/wk; >3/wk	FFQ	ICD-10 (I20-25)	Age, sex, time since diabetes diagnosis, BMI, physical activity, education, cigarette smoking, total energy intake, alcohol, history of high cholesterol, history of hypertension and DASH diet component score

FFQ, Food-frequency questionnaire; BMI, body mass index; ICD, International Classification of Diseases; F, female; M, male; y, year; HDL, High Density Lipoprotein; LDL, Low Density Lipoprotein; ECG, electrocardiogram; SFA, Saturated fatty acids; MUFA, Saturated fatty acids; PUFA, Polyunsaturated fatty acids; CVD, cerebrovascular disease; DASH, Dietary Approaches to Stop Hypertension; wk, week; mo, month; d, day.

## Supplementary Materials

The following are available online at https://www.mdpi.com/2072-6643/12/8/2278/s1, Figure S1: Sensitivity analysis with respect to fish consumption and CHD incidence; Figure S2: Sensitivity analysis with respect to fish consumption and CHD mortality; Figure S3: Funnel plot of the relative risk (RR) of 22 studies on fish consumption and CHD incidence; Figure S4: Funnel plot of the relative risk (RR) of 27 studies on fish consumption and CHD mortality; Table S1: Quality assessment of studies investigating fish consumption and CHD incidence; Table S2: Quality assessment of studies investigating fish consumption and CHD mortality; Table S3: Subgroup analysis and meta-regression analyses for the association between the fish intake and the CHD incidence; Table S4: Subgroup analysis and meta-regression analyses for the association between the fish intake and the CHD mortality.
