# Supplementary material for: Fish Consumption and Coronary Heart Disease: A Meta-Analysis
Source: Nutrients. 2020 Jul 29;12(8):2278. doi: 10.3390/nu12082278 (PMC7468748; doi:10.3390/nu12082278)
Supplement: Supplementary file 1 [file nutrients-12-02278-s001.pdf]

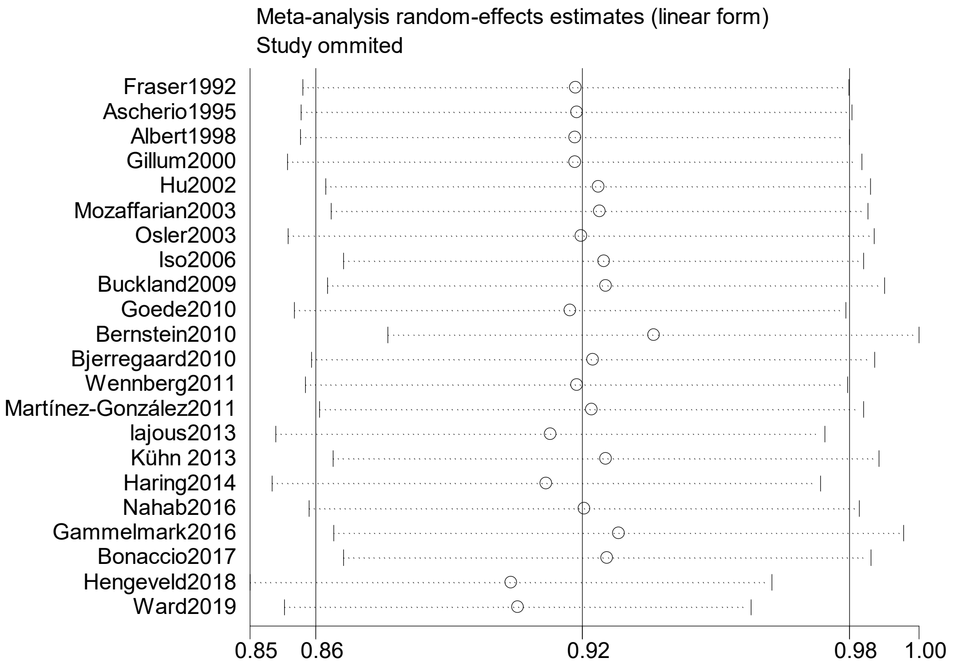


**Figure S1.** Sensitivity analysis with respect to fish consumption and CHD incidence. The circles represent the summary effect after removing one study.

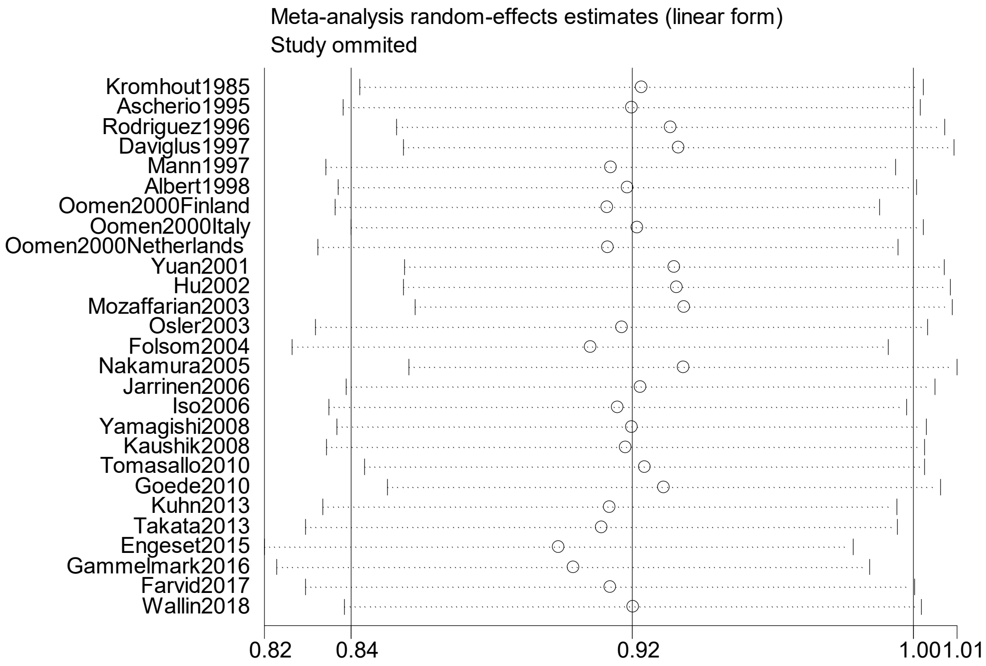


**Figure S2.** Sensitivity analysis with respect to fish consumption and CHD mortality. The circles represent the summary effect after removing one study.

.

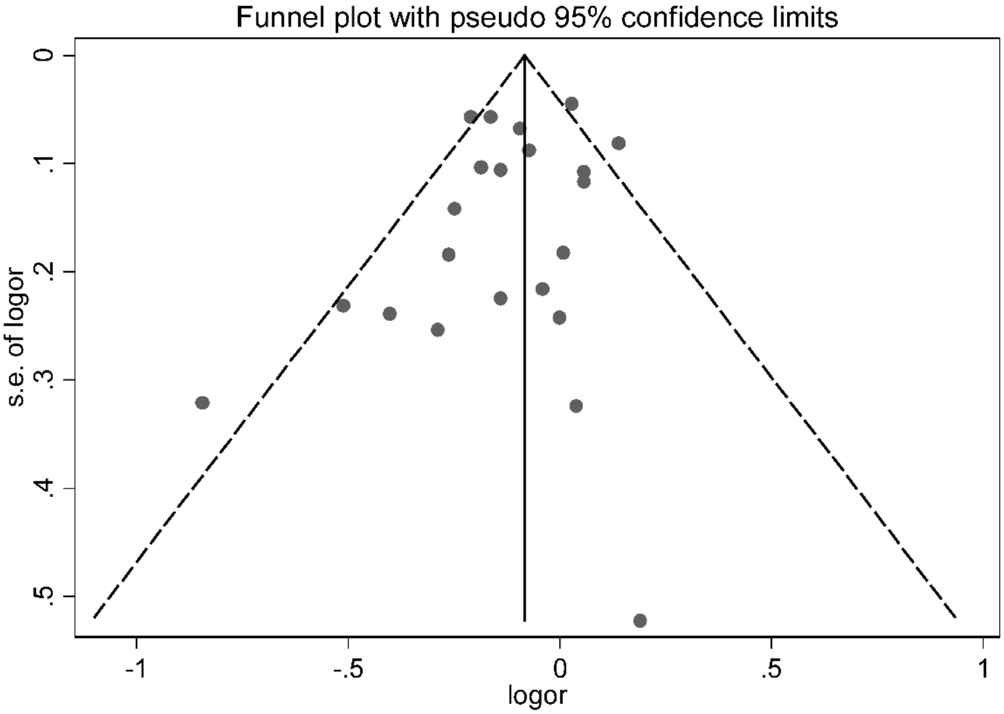


**Figure S3.** Funnel plot of the relative risk (RR) of 22 studies on fish consumption and CHD incidence. Each dot represents a different study.

.

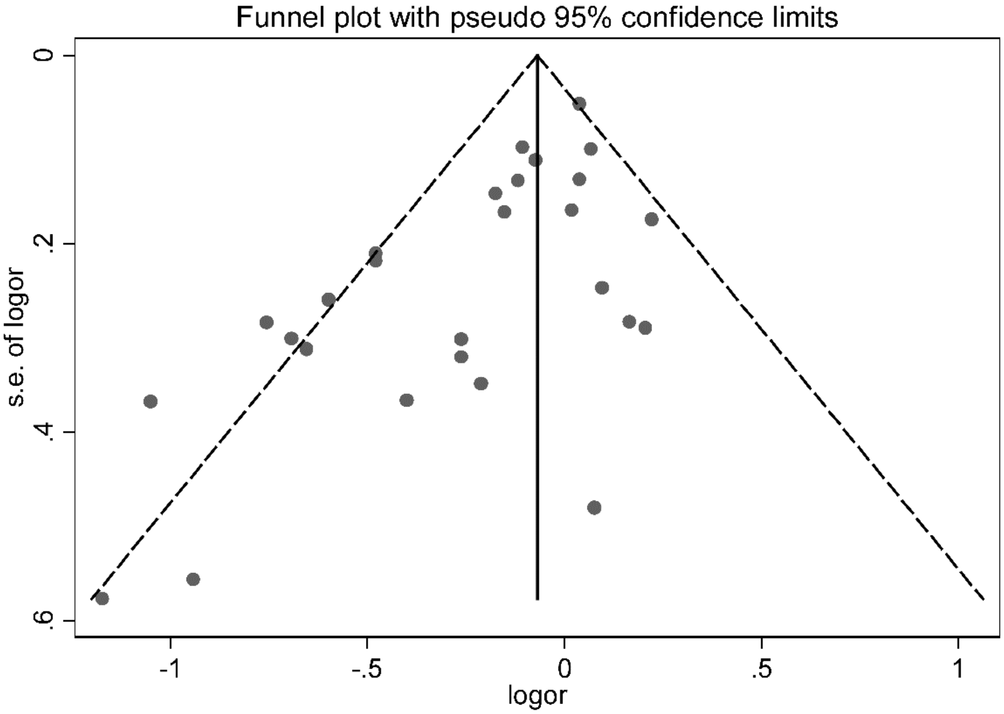


**Figure S4.** Funnel plot of the relative risk (RR) of 27 studies on fish consumption and CHD mortality. Each dot represents a different study.

**Table S1.** Quality assessment of studies investigating fish consumption and CHD incidence.

| Study | Representativeness of the Exposed Cohort | Selection of the Unexposed Cohort | Ascertainment of Exposure | Demonstration That Outcome of Interest at Start of Study | Comparability of Cohorts on the Basis of the Design or Analysis | Outcome Assessment | Follow-Up Long Enough for the Outcomes to Occur | Adequacy of Follow-Up of Cohorts | Total Quality Scores |
|---|---|---|---|---|---|---|---|---|---|
| Fraser | - | ☆ | ☆ | ☆ | ☆ | ☆ | - | - | ☆☆☆☆☆ |
| Ascherio | - | ☆ | ☆ | ☆ | ☆ | ☆ | - | ☆ | ☆☆☆☆☆☆ |
| Albert | - | ☆ | ☆ | ☆ | ☆☆ | ☆ | - | ☆ | ☆☆☆☆☆☆☆ |
| Gillum | ☆ | ☆ | ☆ | ☆ | ☆ | ☆ | ☆ | - | ☆☆☆☆☆☆☆ |
| Hu | - | ☆ | ☆ | ☆ | ☆☆ | ☆ | ☆ | - | ☆☆☆☆☆☆☆ |
| Mozaffarian | ☆ | ☆ | ☆ | ☆ | ☆ | ☆ | - | - | ☆☆☆☆☆☆ |
| Osler | ☆ | ☆ | ☆ | ☆ | ☆ | ☆ | - | - | ☆☆☆☆☆☆ |
| Iso | ☆ | ☆ | ☆ | ☆ | ☆☆ | ☆ | - | - | ☆☆☆☆☆☆☆ |
| Buckland | ☆ | ☆ | ☆ | ☆ | ☆ | ☆ | - | ☆ | ☆☆☆☆☆☆☆ |
| Goede | ☆ | ☆ | ☆ | ☆ | ☆ | ☆ | - | ☆ | ☆☆☆☆☆☆☆ |
| Bernstein | - | ☆ | ☆ | ☆ | ☆ | ☆ | ☆ | - | ☆☆☆☆☆☆ |
| Bjerregaard | ☆ | ☆ | ☆ | ☆ | ☆ | ☆ | - | ☆ | ☆☆☆☆☆☆☆ |
| Wennberg | - | ☆ | ☆ | ☆ | ☆ | ☆ | - | ☆ | ☆☆☆☆☆☆ |
| Martínez-González | - | ☆ | ☆ | ☆ | ☆ | ☆ | - | ☆ | ☆☆☆☆☆☆ |
| lajous | - | ☆ | ☆ | ☆ | ☆☆ | ☆ | ☆ | - | ☆☆☆☆☆☆☆ |

| | | | | | | | | |
|---|---|---|---|---|---|---|---|---|
| Kühn | ☆ | ☆ | ☆ | ☆ | ☆ | ☆ | - | ☆ | ☆☆☆☆☆☆☆ |
| Haring | ☆ | ☆ | ☆ | ☆ | ☆ | ☆ | ☆ | - | ☆☆☆☆☆☆☆ |
| Nahab | - | ☆ | ☆ | ☆ | ☆ | ☆ | - | - | ☆☆☆☆☆ |
| Gammelmark | ☆ | ☆ | ☆ | ☆ | ☆ | ☆ | ☆ | - | ☆☆☆☆☆☆☆ |
| Bonaccio | ☆ | ☆ | ☆ | ☆ | ☆ | ☆ | - | ☆ | ☆☆☆☆☆☆☆ |
| Hengeveld | ☆ | ☆ | ☆ | ☆ | ☆ | ☆ | ☆ | ☆ | ☆☆☆☆☆☆☆☆ |
| Ward | - | ☆ | ☆ | ☆ | ☆ | ☆ | - | - | ☆☆☆☆☆ |

**Table S2.** Quality assessment of studies investigating fish consumption and CHD mortality.

| Study | Representativeness of the Exposed Cohort | Selection of the Unexposed Cohort | Ascertainment of Exposure | Demonstration that Outcome of Interest at Start of Study | Comparability of Cohorts on the Basis of the Design or Analysis | Outcome Assessment | Follow-Up Long Enough for the Outcomes to Occur | Adequacy of Follow-Up of Cohorts | Total Quality Scores |
|---|---|---|---|---|---|---|---|---|---|
| Kromhout | - | ☆ | ☆ | ☆ | ☆☆ | ☆ | ☆ | ☆ | ☆☆☆☆☆☆☆☆ |
| Ascherio | - | ☆ | ☆ | ☆ | ☆ | ☆ | - | ☆ | ☆☆☆☆☆☆ |
| Rodriguez | - | ☆ | ☆ | ☆ | ☆☆ | ☆ | ☆ | - | ☆☆☆☆☆☆☆ |
| Daviglus | - | ☆ | ☆ | ☆ | ☆☆ | ☆ | ☆ | ☆ | ☆☆☆☆☆☆☆☆ |
| Mann | - | - | ☆ | ☆ | ☆ | ☆ | - | - | ☆☆☆☆ |
| Albert | - | ☆ | ☆ | ☆ | ☆☆ | ☆ | - | ☆ | ☆☆☆☆☆☆☆ |
| Oomen | - | ☆ | ☆ | ☆ | ☆ | ☆ | ☆ | ☆ | ☆☆☆☆☆☆☆ |
| Yuan | - | ☆ | ☆ | ☆ | ☆☆ | ☆ | - | ☆ | ☆☆☆☆☆☆☆ |
| Hu | - | ☆ | ☆ | ☆ | ☆☆ | ☆ | ☆ | - | ☆☆☆☆☆☆☆ |
| Osler | ☆ | ☆ | ☆ | ☆ | ☆ | ☆ | - | - | ☆☆☆☆☆☆ |
| Mozaffarian | ☆ | ☆ | ☆ | ☆ | ☆ | ☆ | - | - | ☆☆☆☆☆☆ |
| Folsom | - | ☆ | ☆ | ☆ | ☆☆ | ☆ | ☆ | - | ☆☆☆☆☆☆☆ |
| Nakamura | ☆ | ☆ | ☆ | ☆ | ☆ | ☆ | ☆ | ☆ | ☆☆☆☆☆☆☆☆ |
| Jarvinen | ☆ | ☆ | ☆ | ☆ | ☆ | ☆ | ☆ | - | ☆☆☆☆☆☆☆ |

| | | | | | | | | | |
|---|---|---|---|---|---|---|---|---|---|
| Iso | ☆ | ☆ | ☆ | ☆ | ☆☆ | ☆ | - | - | ☆☆☆☆☆☆☆ |
| Yamagish | - | ☆ | ☆ | ☆ | ☆☆ | ☆ | - | - | ☆☆☆☆☆☆ |
| Kaushik | - | ☆ | ☆ | ☆ | ☆☆ | ☆ | - | - | ☆☆☆☆☆☆ |
| Tomasallo | - | ☆ | ☆ | ☆ | ☆ | ☆ | - | - | ☆☆☆☆☆ |
| Goede | ☆ | ☆ | ☆ | ☆ | ☆ | ☆ | - | ☆ | ☆☆☆☆☆☆☆ |
| Kuhn | ☆ | ☆ | ☆ | ☆ | ☆ | ☆ | - | ☆ | ☆☆☆☆☆☆☆ |
| Takata | - | ☆ | ☆ | ☆ | ☆☆ | ☆ | - | ☆ | ☆☆☆☆☆☆☆ |
| Engeset | ☆ | - | ☆ | ☆ | ☆ | ☆ | ☆ | - | ☆☆☆☆☆☆ |
| Gammelmark | ☆ | ☆ | ☆ | ☆ | ☆ | ☆ | ☆ | - | ☆☆☆☆☆☆☆ |
| Farvid | ☆ | ☆ | ☆ | ☆ | ☆☆ | ☆ | - | ☆ | ☆☆☆☆☆☆☆☆ |
| Wallin | - | - | ☆ | ☆ | ☆☆ | ☆ | ☆ | - | ☆☆☆☆☆☆ |

**Table S3.** Subgroup analysis and meta-regression analyses for the association between the fish intake and the CHD incidence.

| Factors Stratified | NO † | Pooled Relative Risk (95% CI) | Heterogeneity | | *P* [b] |
|---|---|---|---|---|---|
| | | | $I^2$ (%) | *P* [a] | |
| Region | | | | | 0.132 |
| North America | 11 | 0.94(0.85,1.05) | 46.3% | 0.045 | |
| Europe | 10 | 0.89(0.82,0.97) | 42.2% | 0.076 | |
| Asia | 1 | 0.43(0.23,0.81) | - | - | |
| Gender | | | | | 0.102 |
| Male | 8 | 0.96(0.89,1.04) | 3.9% | 0.400 | |
| Female | 8 | 0.85(0.78,0.92) | 5.6% | 0.387 | |
| Follow-up period (years) | | | | | 0.734 |
| ＜10 | 9 | 0.87(0.74,1.02) | 47.1% | 0.057 | |
| ≥10 | 13 | 0.91(0.84,0.99) | 51.5% | 0.016 | |
| Quality score | | | | | 0.880 |
| ≥7 | 13 | 0.90(0.83,0.98) | 50.6% | 0.019 | |
| <7 | 9 | 0.91(0.80,1.03) | 47.1% | 0.057 | |

† Number of included studies; P [a] for heterogeneity; P [b] for meta-regression analysis.

**Table S4.** Subgroup analysis and meta-regression analyses for the association between the fish intake and the CHD mortality.

| Factors Stratified | NO † | Pooled Relative Risk (95% CI) | Heterogeneity | | *P* [b] |
|---|---|---|---|---|---|
| | | | $I^2$ (%) | *P* [a] | |
| Region | | | | | 0.294 |
| Europe | 12 | 0.98(0.87,1.09) | 28.9% | 0.162 | |
| Asia | 6 | 0.81(0.64,1.02) | 51.0% | 0.069 | |
| North America | 8 | 0.65(0.49,0.86) | 54.2% | 0.033 | |
| Australia | 1 | 0.89(0.69,1.16) | - | - | |
| Gender | | | | | 0.826 |
| Male | 13 | 0.88(0.74,1.05) | 57.5% | 0.005 | |
| Female | 6 | 0.87(0.73,1.04) | 37.4% | 0.157 | |
| Follow-up period (years) | | | | | 0.995 |
| ≤10 | 3 | 0.75(0.44,1.30) | 62.2% | 0.071 | |
| 10-20 | 17 | 0.88(0.79,0.99) | 48.7% | 0.013 | |
| ≥20 | 7 | 0.79(0.60,1.04) | 55.9% | 0.034 | |
| Quality score | | | | | 0.828 |
| ≥7 | 18 | 0.83(0.72,0.96) | 53.6% | 0.004 | |
| <7 | 9 | 0.88(0.75,1.02) | 48.5% | 0.049 | |

† Number of included studies; P [a] for heterogeneity; P [b] for meta-regression analysis.
